# Psychosocial Outcomes in Autistic Children Before and During the COVID-19 Pandemic

**DOI:** 10.1007/s10803-023-06101-8

**Published:** 2023-09-10

**Authors:** Rachel Plak, Ralph Rippe, Inge Merkelbach, Sander Begeer

**Affiliations:** 1 Clinical Neurodevelopmental Sciences, Institute of Education and Child Studies, Faculty of Social and Behavioral Sciences, Leiden University, Leiden, The Netherlands; 2 Research Methods and Statistics, Institute of Education and Child Studies, Faculty of Social and Behavioral Sciences, Leiden University, Leiden, The Netherlands; 3 Erasmus School of Social and Behavioural Sciences, Erasmus University Rotterdam, Rotterdam, The Netherlands; 4 Department of Clinical, Neuro- & Developmental Psychology, Faculty of Behavioural and Movement Sciences, Vrije Universiteit Amsterdam, Amsterdam, The Netherlands; 5 Amsterdam Public Health Research Institute, Amsterdam, The Netherlands

**Keywords:** Autism, Children, COVID-19, Pandemic, Psychosocial outcomes, Longitudinal

## Abstract

Studies on the impact of the COVID-19 pandemic on autistic children’s psychosocial outcomes have shown mixed results. In the current study we aimed to gain a better insight into the effect of the COVID-19 pandemic by comparing psychosocial outcomes collected pre-pandemic with data collected during the pandemic. We used the Strengths and Difficulties Questionnaire (SDQ) to examine change over time in psychosocial outcomes of autistic children from pre-pandemic (T0) to lockdown I (T1) and lockdown II (T2) in the Netherlands. We expected a deterioration in psychosocial outcomes. There were 224 participants in T0 and T1, of which 141 also participated in T2. The results showed a surprising improvement in psychosocial outcomes from T0 to T1. Special education and female gender were associated with increased difficulties over time, while higher age was associated with decreased difficulties. At the subdomain level we found that emotional problems remained stable, while hyperactivity, conduct problems, and peer problems decreased, and prosocial behavior increased. Attending special education predicted increased peer problems over time, while higher age predicted both decreased conduct problems and increased prosocial behavior over time. The COVID-19 pandemic may have temporarily improved the fit between the psychosocial needs and the environment for children with autism in the Netherlands.

**Supplementary Information:**

The online version contains supplementary material available at 10.1007/s10803-023-06101-8.

The COVID-19 pandemic introduced several challenges to daily life, such as quarantine, social distancing, and other restrictions to prevent the spread of the virus (Neece et al., 2020). These restrictions likely had a specific impact on autistic children, given their need for structure, routine (American Psychiatric Association, 2013), and community-based support, such as interventions in school (Aishworiya & Kang, 2021). Gaining insight into the responses of autistic children to the COVID-19 pandemic yields knowledge about how they face sudden changes in their daily routine. Lessons from the COVID-19 pandemic may enable us to direct policymakers and clinicians on how to support children with autism. In this study we aimed to gain a better insight into the psychosocial outcomes of autistic children during the pandemic compared to before the pandemic.

There is a silver lining to the pandemic; COVID-19 measures may have led to increased structure in daily life due to social distancing and the elimination of many organized activities. For some autistic children, pandemic-related changes may have induced positive effects (Heyworth et al., 2021). Increased family contact, time to pursue hobbies and calming activities, and a slower pace of life and learning (Ameis et al., 2020; Mutluer et al., 2020; Neece et al., 2020) may improve psychosocial outcomes. However, disruption of daily routines, a lack of certainty, limited access to support or therapy, and increased parental responsibility could increase distress in autistic children (Ameis et al., 2020; Bellomo et al., 2020; Colizzi et al., 2020; Patel et al., 2020; Pellicano et al., 2021). It is very important to delineate the spectrum of consequences of the COVID-19 pandemic for such a highly diverse group as autistic children.^1^

The impact of the COVID-19 pandemic on autistic children’s psychosocial outcomes (clinical characteristics of mental health, wellbeing, and the impact of the pandemic on daily life) has been mixed. Studies have shown increased (Amorim et al., 2020; Colizzi et al., 2020; di Renzo et al., 2020; Linnavalli & Kalland, 2021 Masi et al., 2021; Mutluer et al., 2020; Nuñez et al., 2021; O’Sullivan et al., 2021; Panjwani et al., 2021; Vasa et al., 2021; Fong et al., 2021), stable (Siracusano et al., 2021; Toseeb & Asbury, 2023), or decreased difficulties (Lugo-Marín et al., 2021; Mumbardó-Adam et al., 2021). Most COVID-19 studies of psychosocial outcomes in autistic children started data collection during the pandemic, or retrospectively compared children’s behavior predating and during the pandemic, risking bias in sample selection; parents who had notably very positive or very negative COVID-19-related experiences may have been more likely to participate. In addition, bias can also occur in parents’ reporting on their child’s behavior; it may be difficult for parents to disentangle autism-related difficulties from pandemic-related difficulties. Generally, studies without pre-COVID-19 data—that is, without a clear baseline of children’s psychosocial status—show a worsening of outcomes. Those with pre-COVID-19 data (Mutluer et al., 2020; Lugo-Marín et al., 2021; Toseeb & Asbury, 2023) show mixed results, with stability in adaptive functioning, problematic behaviors, and repetitive behaviors (Siracusano et al., 2021), or even increases in wellbeing *during* compared to before quarantine; the children seemed generally comfortable with the situation and did not long for previous routines (Mumbardó-Adam et al., 2021). In conclusion, methodological variation between studies may contribute to the mixed results for impact of the COVID-19 pandemic. To date, several predictors of pandemic-related changes in psychosocial outcomes in autistic children have been identified. Attending regular education and higher age were related to increased anxiety and depression, while female gender increased depression (Toseeb & Asbury, 2023). Behavior problems of children with autism predating the COVID-19 pandemic increased disruptive behavior, but higher age and living with a single parent decreased disruptive behavior (Colizzi et al., 2020). Child understanding of COVID-19, COVID-19 infection within the family, low family income, and raised parental depression and anxiety symptoms increased mental health problems in autistic children (Vasa et al., 2021). Of these studies, only Toseeb and Asbury (2023) included pre-COVID-19 data.

In the current study we aimed to explore change over time in psychosocial outcomes of autistic children from before the pandemic to during the COVID-19 lockdown in the Netherlands. Using the Strengths and Difficulties Questionnaire (SDQ; Goodman, 1997), we examined changes over three time points. We also examined which child-, family-, and COVID-19-related psychosocial predictors were associated with changes in these psychosocial outcomes. Combining extensive pre-COVID-19 data with newly collected pandemic data from validated questionnaires, we expected an overall deterioration in psychosocial outcomes over time. Based on the previously described literature and to do justice to the heterogeneity of autism, we included the following predictors: autistic traits, age, gender, type of education, co-occurring conditions, pre-existing mental health problems before COVID-19, family composition, COVID-19 family context, including worrying about COVID-19, and socioeconomic status.

## Method

### Design

**Fig. 1:**
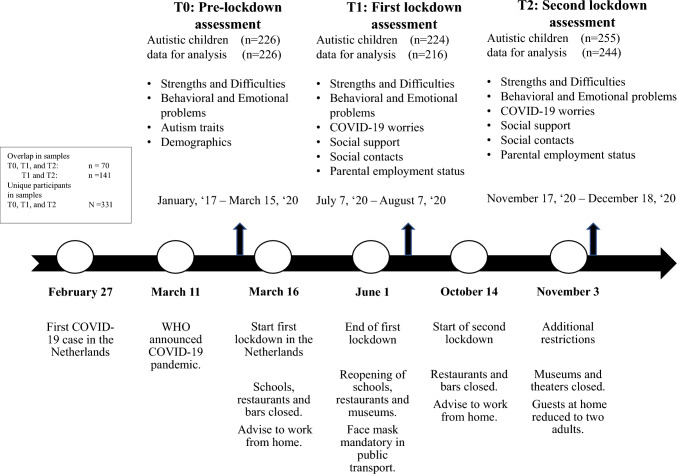
Study design and timeline of COVID-19-Related Restrictions in the Netherlands in 2020. Based on Fig. 1 in Scheeren et al. (2022)

The current study had a longitudinal design, including three online parental reports (T0, T1, T2) of psychosocial outcomes of their autistic child predating and during the COVID-19 pandemic. We examined pre-COVID-19 data derived from the Netherlands Autism Register (NAR) from 2017 to 2020 (T0) in the Netherlands. We collected data in the Netherlands after COVID-19 lockdown I from July 7 to August 7, 2020 (T1) and during lockdown II from November 17 to December 18, 2020 (T2). See Fig. 1 for an overview of the study design and the COVID-19 restrictions in the Netherlands during data collection.

### Participants

Longitudinal data were derived from the NAR (https://www.nederlandsautismeregister.nl), a database of autistic and non-autistic adults and parents and caregivers of autistic individuals, including online parental or caregivers’ reports, as well as sociodemographic data, since 2017. Questionnaires in the current study were completed by parents, caregivers, or legal guardians (hereafter: parents) of children and adolescents with a formal diagnosis of autism (American Psychiatric Association, 2013) made by a qualified clinician not affiliated with the current study. The sample included 224 participants in T1 and 255 in T2. Of the 224 participants in T1, 141 also participated in T2. Therefore, 83 participants only participated in T1 (and not in T2), 114 only participated in T2, and 141 participated in both T1 and T2. See Fig. 1 for an overview of the number of participants per time point.

In all waves we included data of 331 unique participants. Between 2017 and March 2020 (T0), before the first lockdown in the Netherlands, we collected data from parents of autistic children as part of the standard annual online NAR survey (n = 226); of these children, 24% were girls, mean age was 12.4 years, and 58% were in special education. In July 2020 (T1), 216 parents of autistic children (19% girls; mean age 12.8 years; 71% in special education) participated in our COVID-19 study (see Table 1 for sample characteristics). In November and December 2020 (T2), 244 parents once again participated (15% girls; mean age 12.4 years; 64% in special education).

**Table 1:** Descriptive Statistics of Overall Group of Autistic Children with SDQ (Strengths and Difficulties Questionnaire) Measurements Before the COVID-19 Pandemic (T0: 2017–2020), During T1 (July 7, 2020—August 7, 2020), and During T2 (November 17, 2020—December 18, 2020) in the Netherlands

Variable	Level	Total	T0	T1	T2
		n = 331	n = 226	n = 216	n = 244
Gender	Boys	257 (78%)	172 (76%)	174 (81%)	184 (85%)
Age (years)		12.6 (3.8)	12.4 (2.5)	12.8 (3.4)	12.4 (3.8)
Intellectual ability (IQ)	< 86	89 (27%)	25 (11%)	62 (29%)	64 (26%)
	86–115	150 (45%)	116 (51%)	102 (47%)	105 (43%)
	> 115	92 (28%)	85 (38%)	52 (24%)	75 (31%)
Reported ethnicity	Dutch	316 (95%)	225 (99%)	204 (94%)	238 (98%)
	Other	15 (5%)	1 (1%)	12 (6%)	6 (2%)
Type of education	Regular	105 (32%)	94 (42%)	62 (29%)	89 (36%)
	Special /Other	226 (68%)	132 (58%)	154 (71%)	155 (64%)
Mother educational level	Lower	52 (15.7%)	33 (14.6%)	36 (16.7%)	37 (15.2%)
	Middle	96 (29.0%)	69 (30.5%)	68 (31.5%)	67 (27.5%)
	High	183 (55.3%)	124 (54.9%)	112 (51.9%)	140 (57.4%)
Father educational level	Lower	66 (19.9%)	40 (17.7%)	45 (20.8%)	47 (19.3%)
	Middle	108 (32.6%)	78 (34.5%)	80 (37.0%)	80 (32.8%)
	High	157 (47.4%)	108 (47.8%)	91 (42.1%)	117 (47.9%)
Household # of persons		3.0 (0.9)	3.9 (0.9)	3.8 (1.0)	3.8 (0.9)
AQ total score		81.6 (10.8)	81.6 (10.8)	83.0 (10.2)	80.9 (11.2)
Co-occurring mental health condition(s)	Yes	148 (45%)	105 (46%)	106 49%)	105 (43%)
	No	168 (51%)	113 (50%)	98 (45%)	129 (53%)
	Unknown	15 (5%)	8 (4%)	12 6%)	10 (4%)
SDQ total score		16.3 (5.8)	14.5 (6.2)	15.9 (5.6)	15.9 (5.9)
Satisfaction with social contacts		2.0 (1.1)	1.8 (1.0)	2.1 (1.2)	2.1 (1.2)
Social isolation		4.9 (2.6)	NA	4.9 (2.9)	4.8 (2.3)
COVID-19-related worries (child)		4.7 (2.7)	NA	5.0 (2.9)	4.6 (2.5)
COVID-19-related worries (parent)		5.7 (2.2)	NA	5.8 (2.3)	5.6 (2.1)

AQ refers to Autism-Spectrum Quotient. Levels of parental education are defined as Low (*LeerwegOndersteunend Onderwijs* [special needs education], *Praktijkonderwijs* [practical education], *Voortgezet Speciaal Onderwijs* [secondary special education], and all levels of primary school and high school), Middle (*Lager Beroepsonderwijs* [lower secondary vocational education] and *Middelbaar Beroepsonderwijs* [upper secondary vocational education]), and High (*HBO/HTS/HEAO/Lerarenopleiding* [higher professional education/ higher technical education/ higher economic and administrative education/ teacher training] and University: BA/BSc/MA/MSc/MEng)

### Measures

#### Sociodemographic Domain (Pre-COVID-19 Data)

Age, gender, socioeconomic status, and family characteristics were assessed pre-COVID-19. Employment status of parents of autistic children was assessed at T1 and T2; participants indicated whether they currently had paid work, either salaried or self-employed (yes = 1; no = 0).

#### COVID-19 Family Context Domain (Newly Collected Data)

COVID-19 infection within the family, key profession(s) within the family, and parents’ work-from-home status were assessed at T1**.** Internal consistency of single items was not evaluated.

#### Children’s and Parents’ COVID-19-Related Worries (Newly Collected Data)

To assess children’s and parents’ worries about contracting COVID-19, we administered a 6-item questionnaire to parents at T1 and T2. The questionnaire, a modified version of the questionnaire described in Scheeren et al. (2022), asked parents of autistic children to rate their child’s COVID-19-related worries as well as their own. The first items “How worried has your child been about the COVID-19 pandemic in recent weeks?” and “How worried have you been about the COVID-19 pandemic in recent weeks?” were rated on a 10-point scale (1 = *not worried* to 10 = *extremely worried*). Two items describing children’s and parents’ worries about “potentially contracting COVID-19” and “close contacts potentially contracting COVID-19” were each rated on a 5-point scale (1 = *never* to 5 = *always or almost always*). To create a rating out of 10, scores on these two items were multiplied by two and then added to the first item score, yielding a maximum score of 30; higher scores indicate more COVID-19-related worries. Because a low number of items reduces internal consistency, Cronbach’s α estimates were moderate: 0.63 (T1) and 0.61 (T2); McDonald’s ω estimates were 0.64 (T1) and 0.63 (T2).

#### Autistic Traits (Pre-COVID-19 Data)

Autistic traits were measured with the Autism-Spectrum Quotient-Child-28 (AQ-Short; Hoekstra et al., 2011), consisting of 28 items addressing autism severity, social behavior (social skills, routine, switching, and imagination), and fascination for numbers/patterns, using a two-factor model. Prior to the pandemic, participants were invited to respond using a 4-point Likert scale (1 = *totally agree* to 4 = *totally disagree*). Higher scores indicated higher autism severity. In the present study, Cronbach’s α estimates were 0.85 (total score), 0.74 (Social), 0.67 (Routine), 0.58 (Switching), 0.70 (Imagination), and 0.71 (Numbers and Patterns). McDonald’s ω estimates were 0.85 (total score), 0.75 (Social), 0.67 (Routine), 0.60 (Switching), 0.70 (Imagination), and 0.74 (Numbers and Patterns). (See also Appendix C.)

#### Co-occurring Mental Health Conditions (Pre-COVID-19 Data)

The presence of mental health conditions co-occurring with autism in children was determined on the basis of self-reported responses from their parents and coded as 1 (*prior mental health condition*) or 0 (*no prior mental health condition reported*).

#### Sensory Characteristics of the Home Environment (Pre-COVID-19 Data)

Prior to the COVID-19 pandemic, parents reported on various sensory characteristics present in the home situation related to environmental nuisance, using a 7-item questionnaire. Three items regarding the presence of noise nuisance, traffic, and odors/dust/dirt in the neighborhood were developed by Statistics Netherlands. The remaining four items regarding the presence of noises and smells in the immediate vicinity of the house and noises and smells inside the house were developed by the NAR. All items were rated on a 3-point scale (3 = *often*, 2 = *sometimes*, 1 = *never*). In the present study, the Cronbach’s α estimate was 0.74 and McDonald’s ω estimate was 0.75.

### Outcome Measures Indicating Psychosocial Outcomes

#### Psychosocial Outcomes (Including Pre-COVID-19 Data and Newly Collected Data)

Emotional and behavioral problems were measured with the Dutch version of the Strengths and Difficulties Questionnaire (Goodman, 1997, 2001; van Widenfelt et al., 2003). This is a 25-item instrument that assesses difficulties in four subdomains: emotional symptoms, conduct problems, hyperactivity/inattention, and peer problems; and also prosocial behavior. Each item is rated on a 3-point Likert-type scale (0 = *not true*, 1 = *somewhat true*, 2 = *certainly true*). Except for prosocial behavior, a higher score indicates more difficulties. In the current study, Cronbach’s α estimates for the SDQ total score were 0.80 (T0), 0.73 (T1), and 0.78 (T2). For the subdomains, α ranged between 0.52 and 0.79. McDonald’s ω estimates for the SDQ total score were 0.80 (T0), 0.74 (T1) and 0.78 (T2). For the subdomains, ω ranged between 0.54 and 0.80. Details are provided in Appendix C.

#### Social Contacts/Social Wellbeing During the COVID-19 Pandemic (Newly Collected Data)

An adapted version of the Cantril Ladder was used to assess social wellbeing during the COVID-19 pandemic, with two items on an 11-point scale ranging from 0 (*bad)* to 10 (*very good)*, or 0 *(not socially isolated)* to 10 *(extremely socially isolated)*, and one item on a 3-point scale on satisfaction with social contacts, with values 1 (*yes*), 2 (*neutral*), 3 (*no*) and a separate score 4 (*unknown*). As described in Bartels et al. (2012), previous psychometric analysis has shown that the correlation between the latent factor scores of these three measures of subjective wellbeing range between 0.70 and 0.95, indicating that a dimensional score combining these three measures is a valid and reliable measure of overall subjective wellbeing. In the present study, Cronbach’s α estimates for this were 0.32 (T1) and 0.56 (T2). McDonald’s ω estimates were 0.50 (T1) and 0.60 (T2).

#### General Wellbeing During the COVID-19 Pandemic (Newly Collected Data)

The general wellbeing of the child during the COVID-19 pandemic was assessed using a single-item question, an adapted version of the Cantril Ladder (Levin & Currie, 2014). This question was rated on an 11-point scale ranging from 0 (*bad*) to 10 (*very good*). A detailed discussion of the reliability and validity of the adapted version of the Cantril Ladder is provided in Levin and Currie (2014).

### Statistical Approach

The collected data were analyzed in R version 4.0.2. Multilevel regression models were estimated using the lmer and mitml packages to assess changes in (repeated) measures of psychosocial outcomes before and in two waves during the COVID-19 pandemic, to assess changes in psychosocial outcomes between T1 and T2, and to predict wellbeing across timepoints. The intraclass correlation was estimated using nesting of repeated measurements within subjects in the intercept-only model and was found to be high (ICC = 0.63): There are statistically significant differences in overall levels of difficulties between individuals, while difficulty scores within subjects are more similar than difficulty scores between individuals. Therefore, all further models were estimated with a random intercept at subject level. Random slopes were not hypothesized and therefore not tested in the sequence of models. None of the models revealed high correlations between fixed effects. The measure of *worry* about contracting COVID-19 was used (only) for consistency with related work on data from the NAR (Scheeren et al., 2022).

To assess changes over time related to the emergence of COVID-19, we estimated the repeated measures associations using multilevel linear regression models with a random intercept per participant to account for dependencies and to allow for within subject inference beyond the current sample. Random slopes were not hypothesized and therefore not structurally evaluated. In addition, sample size limitations did not allow for further exploration. Models for the SDQ total scores were estimated with both parametric linear effects and nonlinear effects over time. Results were equivalent for both model types. To avoid overidentification or sparse estimations, all results presented are based on parametric linear estimation. To assess robustness of procedures and results, models were fitted using complete cases only, supplemented by using (restricted) maximum likelihood, and pooled estimation on multiply imputed datasets (50 datasets). The two-tailed significance level was set to an alpha of 5%.

## Results

### Sample Descriptives

Summary statistics (means, standard deviation, standardized skewness, % missingness) are shown in Table 1, which describes sociodemographic variables and key constructs for the full available sample at each timepoint.

Strong positive skewness was observed at T_0_ for age, IQ, number of persons in the household, and SDQ coping; and during the pandemic for satisfaction with social contacts, fear of contracting COVID, and being in a key profession, and for the total score of COVID impact on daily life. Negative skewness was observed for a parent being in a key profession. Similar patterns were observed at T1 and T2. However, based on natural skewness, no extreme outlying scores were identified and were therefore not addressed. Given the overall sample size and assuming correct model specification, normality of the sampling distribution is not directly guaranteed but is very probable, according to the central limit theorem (see Fischer, 2011 and Seneta, 2013 for an overview). Therefore, logarithmic transformations (with base 10) were applied only to variables with numerical scales that showed extreme skewness and did not include natural zero values: Impact on Daily Life and Satisfaction with Social Life. Outliers and extreme cases were identified after transformation. Outliers were defined as having a value exceeding 1.5 times the interquartile range (IQR) above or below the median. Extreme cases were defined as having values exceeding 3 times the IQR above or below the median; these are generally candidates for removal. No extreme cases were identified for any of the analysis variables at this stage. The Variance Inflation Factors (VIFs) for all predictors ranged between 1.02 and 1.47 in the separate waves and between 1.05 and 1.32 in all waves combined. As no VIF exceeded the 1.5 threshold, no indication of collinearity was found.

### Missing Values

Missingness structure was assessed using Little’s MCAR test (Little, 1988). The MCAR test for sociodemographic characteristics at pre-COVID-19 observation was not significant (χ^2^ = 25.31, *df* = 17, *p* = 0.089). The MCAR test for AQ and SDQ at T0 was not significant (χ^2^ = 0.85, *df* = 2, *p* = 0.65). The MCAR test for T1 was also not significant (χ^2^ = 5.82, *df* = 2, *p* = 0.054), and again not significant for T2 (χ^2^ = 4.60, *df* = 2, *p* = 0.10). In combination, data points for AQ and SDQ were missing completely at random. Missing values for Social Satisfaction and Impact on Daily Life scores were also missing completely at random (χ^2^ = 5.40, *df* = 6, *p* = 0.49).

Full results for psychosocial outcomes at baseline are presented in Appendix A. Participants included only at T1 differed from baseline scores for participants included at all time points solely for SDQ total scores (participants at T1 scored lower, related to the main hypothesis), *t*(74) = 2.09, *p* = 0.040, and for Age (participants at T1 had a lower age), *t*(56) = 2.76, *p* = 0.008.

Participants included only at T2 differed from baseline scores for participants included at all time points solely for SDQ total scores (participants at T2 scored lower, related to the main hypothesis), *t*(136) = 2.43, *p* = 0.017, for Type of Education (*OR* = 0.43, *p* = 0.013; participants included only at T2 had a lower ratio of special education versus regular education), and for Age (participants at T2 had a lower age), *t*(156) = 4.14, *p* < 0.001.

Results from either maximum likelihood or multiple imputation are therefore suitable for additional evaluation of robustness. The number of preselected complete cases differs per hypothesis and per estimated model, ranging between 180 and 331. A total of 331 participants were therefore subjected to model estimation via maximum likelihood and multiple imputation.

### Changes in Wellbeing During the COVID-19 Pandemic

#### Model Selection

Models were evaluated incrementally and compared for fit. Table 2 presents a list of fitted models and their results. Due to the exploratory nature of the dependency structure, each model is described separately. Subsequently, the best fitting and most parsimonious model was selected and then interpreted in detail.

**Table 2:** Results for all fitted multilevel models; dependent variable: SDQ total difficulties score

Model	Effect	FE	SE	*p*	RE σ^2^	Residual σ^2^
1	IntraClass correlation	.63			21.14	12.45
2	time	− 1.14	0.20	< .001	22.21	11.09
3	Time	− 1.12	0.20	< .001	22.11	11.02
	Type of education	1.17	0.54			
4	time	− 1.20	0.37	.001	22.10	11.05
	Type of education	0.95	1.13	.401		
	Time* type of education	0.10	0.45	.816		
5	time	− 1.23	0.20	< .001	20.51	10.88
	Type of education	1.33	0.53	.013		
	Gender	1.64	0.70	.020		
	Age	− 0.32	0.076	< .001		
6	Time	− 1.26	0.25	< .001	21.30	10.54
	Type of education	0.55	0.74	.457		
	Gender	0.52	0.99	.127		
	Age	− 0.39	0.10	< .001		
	IQ	− 0.32	0.57	.57		
	Educational level	− 0.70	0.53	.19		
	Key profession (at least one parent)	0.91	0.88	.30		
7a	Time	0.11	0.30	.69	22.06	6.59
	Type of Education	2.21	0.56	< .001		
	Gender	1.41	0.70	.047		
	Age	− 0.37	0.08	< .001		
	COVID-19-related worries (child)	0.39	0.09	< .001		
	COVID-19-related worries (parent)	− 0.08	0.12	.48		
7b	Time	0.08	0.30	.78	22.84	6.53
	Type of Education	2.08	0.57	< .000		
	Gender	1.41	0.71	.047		
	Age	− 0.37	0.08	< .001		
	Mean score for child/parent COVID-19-related worries	0.38	0.12	.001		
7c	Time	0.13	0.30	.97	22.09	6.59
	Type of Education	2.19	0.56	< .000		
	Gender	1.39	0.70	.047		
	Age	− 0.37	0.077	< .001		
	COVID-19-related worries (child)	0.37	0.088	< .001		
7d	Time	− 0.04	0.29	.88	23.76	6.50
	Type of Education	2.02	0.57	< .001		
	Gender	1.53	0.72	.033		
	Age	− 0.35	0.08	< .001		
	COVID-19-related worries (parent)	0.10	0.11	.35		
8	Time	− 2.42	0.48	< .001	19.96	10.73
	Type of Education	0.55	0.74	.46		
	Gender	1.24	0.91	.17		
	Age	− 0.16	0.11	.13		
	Social satisfaction	0.79	0.27	.003		
9	Time	− 0.05	0.30	.86	22.94	6.69
	Type of education	2.16	0.60	< .001		
	Gender	1.62	0.71	.023		
	Age	− 0.34	0.08	< .001		
	Social isolation	0.18	0.08	.028		

*FE* Fixed Effect, *RE* Random Effect, *SE* Standard Error

First, we fitted a random intercept-only model (model 1), which indicated a non-ignorable nesting and thus the need for multilevel models based on the high intraclass coefficient of 0.63. Model 2 fitted an additional fixed effect of time, showing a significant fit improvement compared to model 1 (χ^2^ = 29.77, *df* = 1, *p* < 0.001). Model 3 improved significantly compared to model 2 (χ^2^ = 4.67, *df* = 1, p = 0.031) by adding a parameter for type of education (special vs. regular). Model 4 elaborated on model 3 by adding the interaction between time and type of education. This addition was not significant (χ^2^ = 0.06, *df* = 1, *p* = 0.815). Therefore the interaction between time and type of education was dropped from subsequent models, and we continued our explorations in comparison to model 3.

Model 5 expanded on model 3 by adding estimations of two demographic covariates for gender and age. Model 5 fitted significantly better compared to model 3 (χ^2^ = 12.13, *df* = 2, *p* = 0.002), and all included main effects were significant. Additions in model 6 of IQ, paternal educational level and having a key profession were not significant (χ^2^ = 3.74, *df* = 3, *p* = 0.29). Therefore IQ, paternal educational level, and having a key profession were dropped from subsequent models, and we continued our explorations in comparison to model 5.

Model 7 expanded considerably on model 5 by additionally estimating an effect for “worry about contracting COVID-19” (χ^2^ = 17.72, *df* = 1, *p* < 0.001). Model 7a adjusted for child and parent worries separately; model 7b adjusted for the mean of the child and parent worries as a single covariate; model 7c adjusted for worries of the child only; model 7d adjusts for worries of the parent only. The results showed a significant effect of “worry”, but due to (too) strong collinearity with time, the effect of time disappeared. Worry about contracting COVID-19 was therefore dropped from the model and model 5 remained the reference model.

Alternatively, in model 8 the additional contribution of social satisfaction compared to model 5 was significant (χ^2^ = 8.34, *df* = 1, *p* = 0.003); however social satisfaction was also collinear with both age and type of education, thereby rendering the overall model non-significant. Thus, social satisfaction was dropped from the model. The same pattern was observed for social isolation in model 9 (χ^2^ = 4.74, *df* = 1, *p* = 0.029), and it was dropped from the model due to collinearity.

#### Final Model Interpretation

Based on the discussion of the results in Table 2, model 5 was selected for final evaluation and interpretation. The fixed effect associations are presented in Table 3.

**Table 3:** Fixed effects of selected final multilevel model; outcome: SDQ total difficulties score

Effect parameter	*B*	*SE*	*p*
Time	− 1.23	0.20	< .001
Type of Education	1.33	0.53	.013
Gender	1.64	0.70	.020
Age	− 0.32	0.08	< .001

**Fig. 2:**
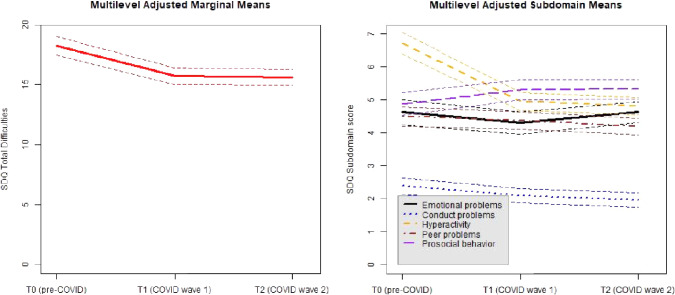
SDQ score profiles over time. Left panel shows the SDQ total difficulty score (range 0–20). Right panel shows the individual SDQ subdomain scores (range 0–7)

Results for SDQ total difficulties indicated a surprising improvement over time. The total difficulties decreased significantly over time (*B* = -1.23, *SE* = 0.20, *p* < 0.001). Furthermore, special education (*B* = 1.32, *SE* = 0.54, *p* = 0.013) and female gender (B = 1.64, SE = 0.70, p = 0.020) were associated with increased SDQ total scores over time. Higher age is associated with a decrease in SDQ total scores over time (*B* = -0.32, *SE* = 0.076, *p* < 0.001). The adjusted effect over time is presented in the left panel in Fig. 2. The driving difficulty subdomains seem to be hyperactivity and conduct problems; both hyperactivity and conduct problems decreased significantly over time, after adjustment for type of education, gender, and age. A similar but weaker decrease was found for peer problems. Higher age was associated with fewer difficulties over time, and girls showed more emotional problems over time compared to boys.

Emotional problems did not change over time when accounting for differences in type of education, gender, and age. However, when additionally—and exploratively—accounting for COVID-19-related worries of the child and the parent, a significant increase in emotional problems over time was observed. This pattern was observed a) when the child and parent worry rating were included as separate predictors, b) when their mean score was included as a predictor, and c) when only the child’s worries were included. However, including only parent worries did not yield any changes to the effect of time compared to the model without COVID-19-related worries. Gender is a significant covariate, with females scoring higher compared to males. Prosocial behavior showed a significant increase over time. This effect is no longer present after adjusting for the child’s and parents’ worry rating.

Results for each individual SDQ subdomain for the same model as selected for the SDQ total difficulties scale are presented in Appendix B. The right panel of Fig. 2 provides a visual overview of the effects of time for each individual subdomain.

A more sensitive and precise determination of different latent profiles could provide more detailed insights but this is beyond the scope of the current paper.

Conclusions did not differ between estimations using preselected complete cases, maximum likelihood estimation, and multiple imputation.

## Discussion

In the current study we examined change over time in psychosocial outcomes in children with autism during the COVID-19 pandemic compared to before the pandemic in the Netherlands. Autistic children showed an unexpected overall improvement in psychosocial outcomes over time, from pre-pandemic to the first lockdown, although the difference between the first and second lockdown was small. Improvements were observed in the SDQ subdomains, with hyperactivity, behavioral problems, and peer problems decreasing; however emotional problems remained stable. Finally, prosocial behavior increased over time. In addition, we found that both special education and female gender predicted an overall increase in difficulties over time, while higher age predicted an overall decrease in difficulties over time. At the SDQ subdomain level, higher age was a predictor of a decrease in conduct problems over time as well as an increase in prosocial behavior over time; attending special education was a predictor of increased peer problems over time. The overall improvement in psychosocial outcomes in children with autism during the pandemic is (partially) in line with some previous findings (Mumbardó-Adam et al., 2021) but contradicts several others (Amorim et al., 2020; Colizzi et al., 2020; di Renzo et al., 2020; Linnavalli & Kalland, 2021; Masi et al., 2021; Mutluer et al., 2020; Nuñez et al., 2021.; O’Sullivan et al., 2021; Panjwani et al., 2021; Vasa et al., 2021; Fong et al., 2021). This may be explained by the availability of pre-COVID-19 data, which may prevent biased parental memory of children’s pre-pandemic behavior.

Although we found an overall improvement in autistic children, at the subdomain level we found stability in emotional problems, such as having worries, being unhappy or downhearted, and being nervous in new situations. These emotional problems predated the COVID-19 pandemic and remained stable during the pandemic, in accordance with Siracusano et al. (2021), who found clinical stability in problematic behavior. Such behaviors generally necessitate intensive intervention in order to change (Siracusano et al., 2021). Furthermore, some aspects of the emotional problems construct, such as worrying and being nervous in new situations, reflect a form of anxiety. About 40% of autistic children are affected by co-occurring anxiety disorder (van Steensel et al., 2011) and symptoms may overlap or interact with autism traits (Shephard et al., 2018). Such fundamental characteristics are not expected to decrease as a result of the pandemic. We found that when children had COVID-19-related worries, this resulted in an increase in emotional problems over time. This finding is in line with Rothe et al. (2021), who found that potential health risks negatively affected emotions in children and adolescents with mental health conditions, including autism. Additionally, we found that when parents were worried this did not affect the child’s level of emotional problems over time. Parents’ COVID-19-related worries may not be directly related to the child’s level of emotional problems.

Some adjustments as a result of the COVID-19 measures may be in line with the needs of some autistic children (e.g., clear perspectives for action in (social) situations, and limited in-person social interactions), resulting in fewer psychosocial difficulties during the pandemic. The absence of usual stressors during the pandemic may have reduced stress in autistic children and consequently psychosocial difficulties, such as hyperactivity. Behaviors like restlessness, fidgeting, being distracted, and failing to finish tasks decreased, contradicting previously found increases in hyperactivity, which were attributed to the absence of planned routines (di Renzo et al., 2020). However, the absence of planned routines could also explain a decrease in hyperactivity (Leitner, 2014): Fewer organized activities and planned routines result in fewer demands in terms of attention and concentration; also, hyperactivity may have been less visible or disruptive in this context. The same mechanism may explain our finding of decreased conduct problems in autistic children during the pandemic.

When autistic children are allowed to organize their own day, they may encounter fewer challenges (e.g., demanding interactions with peers or sensory overload in the school environment), resulting in fewer conduct problems. A slower pace of life (Ameis et al., 2020; Mutluer et al., 2020) during the pandemic might explain the finding of a decrease in conduct problems, such as fighting, being disobedient, and having tantrums. Age predicted conduct problems over time: Older participants showed fewer conduct problems during the pandemic compared to younger participants. Older children are more able to regulate their behavior and emotions than younger children, due to more developed executive functions (Orben et al., 2020). A better understanding of changes in the environment may contribute to fewer conduct problems; possibly this understanding induces a strong sense of control over daily activities and therefore fewer negative emotions, while younger children experience this less and cannot foresee what the day will bring.

During lockdown, children with autism had the opportunity to spend extra time on focused interests, which are associated with wellbeing and satisfaction (Grove et al., 2018). Spending time on activities that bring joy may have contributed to the wellbeing of autistic children during the pandemic.

The pandemic may have encouraged children to seek peer interaction that best suits *their* individual needs, such as interacting with peers online. Despite the overall decrease in peer problems, children attending special education were more likely to show more peer problems than those of the same age attending regular education. Interestingly, over time children with autism showed increased prosocial behavior, despite having fewer opportunities for prosocial actions during the pandemic (van de Groep et al., 2020). This finding is inconsistent with Nonweiler et al. (2020), who found that children with autism showed a decrease in prosocial behavior compared to before the COVID-19 pandemic. There may have been fewer expectations from the environment, allowing autistic children to shift their gaze from their inner world to their environment and thus encouraging behavior that is helpful to others.

Age predicted prosocial behavior over time; older participants showed more prosocial behavior during the pandemic than younger participants. Improvement of cognitive functions in neurotypical adolescents enables young people to better understand the minds of others and take others’ perspectives (Dumontheil et al., 2010). This may also be true for autistic adolescents.

The overall improvement of psychosocial outcomes in autistic children in our sample may partly be attributed to the fact that mental health care in the Netherlands (which is almost fully reimbursed by basic insurance, including tele-health) was mostly continued during the pandemic, preventing the emergence of new problems due to lack of care. This continuation of care may not have been possible in all countries, for example in countries with fewer (digital) mental health facilities and opportunities.

## Limitations

The current study was not without limitations. Parents of autistic children in the United States reported changes in behaviors during the COVID-19 pandemic, particularly in families with low income and food insecurity, thus highlighting the effects of existing disparities on autistic children and their families during the pandemic (Panjwani et al., 2021). Due to a relatively high socioeconomic status (SES) of the participants in this sample, the results of the present study may not be fully generalizable to families with lower SES. Children and adolescents from lower SES households may be more at risk of deterioration of psychosocial outcomes; being housebound may be more manageable when there is plenty of food, personal space in the home for all family members, and time and financial resources for personal growth and enjoying family time. Additionally, the COVID-19 pandemic mainly led to financial stress in the low SES segments of society, potentially negatively influencing family interactions (Spinelli et al., 2021). Future studies should explore socioeconomic factors impacting behavioral changes during uncertain times, such as the COVID-19 pandemic.

Furthermore, the data were derived from parental reports regarding the behavior of their autistic child. Although parents can be reliable informants with regard to their child's behavior, parents’ interpretations may have influenced the results. Additionally, T1 can be partly considered retrospective, since parents were asked to report their child's behavior of several weeks prior. Parents undoubtedly had little trouble remembering their child's behavior from the recent past, but this should be taken into consideration when interpreting the results. In addition, it is possible that some improvements in psychosocial outcomes may be related not only to the changed environment due to COVID-19, but possibly also to a natural process of aging. Finally, some participants took part for the first time in T2 and pre-COVID-19 data may be lacking for some of those participants.

## Future Directions

Lower environmental demands may explain the improved psychosocial outcomes in autistic children and adolescents. If reduced demands result in fewer problems, the pandemic highlights the profound needs of many individuals with autism and their vulnerability to exclusion when exposed to environments designed for neurotypical individuals (Lord et al., 2022). Adapting environmental demands, including their feasibility and desirability, may be beneficial for children with autism. In addition, as a society we need to ask ourselves whether these environmental demands originate from a neurotypical point of view and if these demands are or even should be applicable to neurodiverse young people. We urge clinicians to carefully re-evaluate pandemic experiences together with the child and their family system; what might have worked for them in a pandemic context and what they can take from this to apply outside a pandemic context. For example, granting children more flexibility in their daily routines may provide them with more control to meet their personal needs. Being relieved of environmental demands might give children with autism opportunities to thrive.

The absence of usual stressors during the pandemic, such as limited in-person social interaction, may have contributed to improvement in psychosocial outcomes. In non-pandemic contexts these stressors are present, and clinicians and policymakers should give attention to them by offering targeted support and resources in various settings, such as school, home, and with peers. The adaptations made during the pandemic, including remote support, might have been beneficial for some autistic children. We therefore propose a care system offering remote and hybrid support that can be tailored to specific needs of children with autism and their families.

This longitudinal study includes data collected both before and during the COVID-19 pandemic, reducing bias in sample selection and parental reporting. It provides insights into how adaptive care and increased autonomy during the pandemic may have contributed to positive psychosocial outcomes of autistic children. The robust Dutch welfare system, which includes a social security system covering all citizens, demonstrated high adequacy to deal with the pandemic (Pereirinha & Pereira, 2021). High social expenditure aimed at reducing social exclusion, safety net measures, employment protection, and remote work facilitated by public policies (Pereirinha & Pereira, 2021) in various professional fields, including mental health care, helped mitigate some negative impacts of the pandemic. Our findings of improved psychosocial outcomes contrast somewhat with the stability found in the Italian study of Siracusano et al. (2021), which incorporated data collected before and during the pandemic. However, they align with the Spanish results of Mumbardó-Adam et al. (2021), who also incorporated pre- and during-pandemic data. Southern European countries, including Spain, may be less able to mitigate the negative effects than countries with a welfare system similar to the Dutch, due to higher poverty rates and income inequality. Consequently, Spain as well as Italy required urgent measures to prevent social hardship and protect vulnerable groups (Pereirinha & Pereira, 2021). This may have created a more suitable environment for autistic children, which explains why psychosocial outcomes did not worsen significantly in Italian autistic children (Siracusano et al., 2021) and improved in Spanish autistic children: Their parents reported having more time to teach them new skills, such as autonomy; adopting new strategies to structure their days; and having access to psychological support (Mumbardó-Adam et al., 2021). The environmental changes may have contributed to the reduction of psychopathological symptoms found in another group of Spanish autistic children, although this only reached statistical significance for anxiety, somatization, and obsessive–compulsive domains (Lugo-Marín et al., 2021).

A particular factor contributing to the above findings could be the different living conditions. In the Netherlands and Spain, overcrowding is uncommon (4.8% and 5.9% respectively), compared to Italy (28.3%, Appolloni & D’Alessandro, 2021). More living space per person allows autistic children to retreat from social interactions as needed, which may have improved their psychosocial outcomes during the pandemic.

The improvement seen in Dutch autistic children may not be unique, but is perhaps more likely in similar welfare states, possibly due to urgent measures against social hardship. This underscores not only the variations, severity, and complexity of the COVID-19 contexts worldwide, but may also reflect the range of pandemic responses among autistic children and adolescents. To provide personalized care, it is vital to understand the context and factors—welfare, child, family, housing, or cultural—contributing to these improvements. This should be further explored in future research.

The pandemic may have temporarily made the environment more suitable for children with autism in the Netherlands, improving their psychosocial outcomes. The findings of the current study emphasize the importance of addressing risk of inequality for children with autism.

## Supplementary Information

Below is the link to the electronic supplementary material.

Supplementary file1 (DOCX 24 kb)

## Footnotes

1 Note: the preference for using the term ‘person with autism’ (PFL, person-first language) or ‘autistic person’ (IFL, identity-first language) varies (Buijsman et al., 2022; Lord et al., 2022). We therefore chose to use both terms interchangeably.

## References

[CR1] Aishworiya, R., & Kang, Y. Q. (2021). Including children with developmental disabilities in the equation during this COVID-19 pandemic. *Journal of Autism and Developmental Disorders,**51*(6), 2155–2158. 10.1007/S10803-020-04670-6 32816170 10.1007/s10803-020-04670-6 PMC7438977

[CR2] Ameis, S. H., Lai, M. C., Mulsant, B. H., & Szatmari, P. (2020). Coping, fostering resilience, and driving care innovation for autistic people and their families during the COVID-19 pandemic and beyond. *Molecular Autism,**11*(1), 1–9. 10.1186/s13229-020-00365-y 32698850 10.1186/s13229-020-00365-y PMC7374665

[CR3] American Psychiatric Association. (2013). *Diagnostic and statistical manual of mental disorders* (5th ed.). American Psychiatric Association.

[CR4] Amorim, R., Catarino, S., Miragaia, P., Ferreras, C., & Viana, V. (2020). The impact of COVID-19 on children with autism spectrum disorder. *Revista de Neurología*. 10.33588/rn.7108.2020381 33034366 10.33588/rn.7108.2020381

[CR5] Appolloni, L., & D’alessandro, D. (2021). Housing spaces in nine European countries: A comparison of dimensional requirements. *International Journal of Environmental Research and Public Health,**18*(8), 4278. 10.3390/ijerph18084278 33920693 10.3390/ijerph18084278 PMC8073340

[CR6] Bartels, M., de Moor, M. H. M., van der Aa, N., Boomsma, D. I., & de Geus, E. J. C. (2012). Regular exercise, subjective wellbeing, and internalizing problems in adolescence: Causality or genetic pleiotropy? *Frontiers in Genetics*. 10.3389/fgene.2012.00004 22303410 10.3389/fgene.2012.00004 PMC3261428

[CR7] Bellomo, T. R., Prasad, S., Munzer, T., & Laventhal, N. (2020). The impact of the COVID-19 pandemic on children with autism spectrum disorders. *Journal of Pediatric Rehabilitation Medicine: An Interdisciplinary Approach,**13*, 349–354. 10.3233/prm-200740 10.3233/PRM-200740 32986631

[CR8] Buijsman, R., Begeer, S., & Scheeren, A. M. (2022). “Autistic person” or “person with autism”? Person-first language preference in Dutch adults with autism and parents. *Autism*. 10.1177/13623613221117914 35957517 10.1177/13623613221117914 PMC10074744

[CR9] Colizzi, M., Sironi, E., Antonini, F., Ciceri, M. L., Bovo, C., & Zoccante, L. (2020). Psychosocial and behavioral impact of COVID-19 in autism spectrum disorder: An online parent survey. *Brain Sciences*. 10.3390/brainsci10060341 32503172 10.3390/brainsci10060341 PMC7349059

[CR10] di Renzo, M., di Castelbianco, F. B., Vanadia, E., Petrillo, M., D’Errico, S., Racinaro, L., & Rea, M. (2020). Parent-reported behavioural changes in children with autism spectrum disorder during the COVID-19 lockdown in Italy. *Continuity in Education,**1*(1), 117–125. 10.5334/cie.20 38774533 10.5334/cie.20 PMC11104382

[CR11] Dumontheil, I., Apperly, I. A., & Blakemore, S. J. (2010). Online usage of theory of mind continues to develop in late adolescence. *Developmental Science,**13*(2), 331–338. 10.1111/j.1467-7687.2009.00888.x 20136929 10.1111/j.1467-7687.2009.00888.x

[CR12] Fischer, H. (2011). *A history of the central limit theorem*. Springer. 10.1007/978-0-387-87857-7

[CR46] Fong, H. X., Cornish, K., Kirk, H., Ilias, K., Shaikh, M. F., & Golden, K. J. (2021). Impact of the COVID-19 lockdown in Malaysia: An examination of the psychological well-being of parent-child dyads and child behavior in families with children on the autism spectrum. *Frontiers in Psychiatry*, *12*, 733905. 10.3389/fpsyt.2021.733905 10.3389/fpsyt.2021.733905 PMC8555492 34721108

[CR13] Goodman, R. (1997). The Strengths and Difficulties Questionnaire: A research note. *Journal of Child Psychology and Psychiatry and Allied Disciplines,**38*(5), 581–586. 10.1111/j.1469-7610.1997.tb01545.x 9255702 10.1111/j.1469-7610.1997.tb01545.x

[CR14] Goodman, R. (2001). Psychometric properties of the Strengths and Difficulties Questionnaire. *Journal of the American Academy of Child & Adolescent Psychiatry,**40*(11), 1337–1345. 10.1097/00004583-200111000-00015 11699809 10.1097/00004583-200111000-00015

[CR15] Grove, R., Hoekstra, R. A., Wierda, M., & Begeer, S. (2018). Special interests and subjective wellbeing in autistic adults. *Autism Research,**11*(5), 766–775. 10.1002/aur.1931 29427546 10.1002/aur.1931

[CR16] Heyworth, M., Brett, S., den Houting, J., Magiati, I., Steward, R., Urbanowicz, A., Stears, M., & Pellicano, E. (2021). “It just fits my needs better”: Autistic students and parents’ experiences of learning from home during the early phase of the COVID-19 pandemic. *Autism & Developmental Language Impairments*. 10.1177/23969415211057681 10.1177/23969415211057681 PMC9620701 36381526

[CR17] Hoekstra, R. A., Vinkhuyzen, A. A. E., Wheelwright, S., Bartels, M., Boomsma, D. I., Baron-Cohen, S., Posthuma, D., & van der Sluis, S. (2011). The construction and validation of an abridged version of the autism-spectrum quotient (AQ-Short). *Journal of Autism and Developmental Disorders*. 10.1007/s10803-010-1073-0 20697795 10.1007/s10803-010-1073-0 PMC3076581

[CR18] Leitner, Y. (2014). The co-occurrence of autism and attention deficit hyperactivity disorder in children - What do we know? *Frontiers in Human Neuroscience,**8*(1 APR), 268. 10.3389/fnhum.2014.00268 24808851 10.3389/fnhum.2014.00268 PMC4010758

[CR19] Levin, K., & Currie, C. (2014). Reliability and Validity of an Adapted Version of the Cantril Ladder for Use with Adolescent Samples. *Social Indicators Research*. 10.1007/s11205-013-0507-4

[CR20] Linnavalli, T., & Kalland, M. (2021). Impact of COVID-19 restrictions on the social-emotional wellbeing of preschool children and their families. *Education Sciences,**11*(8), 435. 10.3390/educsci11080435

[CR21] Little, R. J. A. (1988). A test of missing completely at random for multivariate data with missing values. *Journal of the American Statistical Association,**83*(404), 1198–1202. 10.1080/01621459.1988.10478722

[CR22] Lord, Catherine, Charman, Tony, Havdahl, Alexandra, Carbone, Paul, Anagnostou, Evdokia, Boyd, Brian, Carr, Themba, de Vries, Petrus J., Dissanayake, Cheryl, Divan, Gauri, Freitag, Christine M., Gotelli, Marina M., Kasari, Connie, Knapp, Martin, Mundy, Peter, Plank, Alex, Scahill, Lawrence, Servili, Chiara, Shattuck, Paul, … McCauley, James B. (2022). The Lancet Commission on the future of care and clinical research in autism. *The Lancet,**399*(10321), 271–334. 10.1016/S0140-6736(21)01541-5 10.1016/S0140-6736(21)01541-5 34883054

[CR23] Lugo-Marín, J., Gisbert-Gustemps, L., Setien-Ramos, I., Español-Martín, G., Ibañez-Jimenez, P., Forner-Puntonet, M., Arteaga-Henríquez, G., Soriano-Día, A., Duque-Yemail, J. D., & Ramos-Quiroga, J. A. (2021). COVID-19 pandemic effects in people with Autism Spectrum Disorder and their caregivers: Evaluation of social distancing and lockdown impact on mental health and general status. *Research in Autism Spectrum Disorders*. 10.1016/j.rasd.2021.101757 33649707 10.1016/j.rasd.2021.101757 PMC7904459

[CR24] Masi, A., Mendoza Diaz, A., Tully, L., Azim, S. I., Woolfenden, S., Efron, D., & Eapen, V. (2021). Impact of the COVID-19 pandemic on the well-being of children with neurodevelopmental disabilities and their parents. *Journal of Paediatrics and Child Health,**57*(5), 631–636. 10.1111/jpc.15285 33426739 10.1111/jpc.15285 PMC8014782

[CR25] Mumbardó-Adam, C., Barnet-López, S., & Balboni, G. (2021). How have youth with Autism Spectrum Disorder managed quarantine derived from COVID-19 pandemic? An approach to families perspectives. *Research in Developmental Disabilities,**110*, 103860. 10.1016/j.ridd.2021.103860 33486395 10.1016/j.ridd.2021.103860 PMC9758011

[CR26] Mutluer, T., Doenyas, C., & Aslan Genc, H. (2020). Behavioral implications of the Covid-19 process for autism spectrum disorder, and individuals’ comprehension of and reactions to the pandemic conditions. *Frontiers in Psychiatry,**11*, 1263. 10.3389/fpsyt.2020.561882 10.3389/fpsyt.2020.561882 PMC7701051 33304279

[CR27] Neece, C., McIntyre, L. L., & Fenning, R. (2020). Examining the impact of COVID-19 in ethnically diverse families with young children with intellectual and developmental disabilities. *Journal of Intellectual Disability Research*. 10.1111/jir.12769 32808424 10.1111/jir.12769 PMC7461180

[CR28] Nonweiler, J., Rattray, F., Baulcomb, J., Happé, F., & Absoud, M. (2020). Prevalence and associated factors of emotional and behavioural difficulties during COVID-19 pandemic in children with neurodevelopmental disorders. *Children*. 10.3390/children7090128 32899799 10.3390/children7090128 PMC7552706

[CR29] Nuñez, A., Le Roy, C., Coelho-Medeiros, E., & López-Espejo, M. (2021). Factors affecting the behavior of children with ASD during the first outbreak of the COVID-19 pandemic. *Neurological Sciences*. 10.1007/s10072-021-05147-9 33641028 10.1007/s10072-021-05147-9 PMC7914113

[CR47] Orben, A., Tomova, L., & Blakemore, S. J. (2020). The effects of social deprivation on adolescent development and mental health. *The Lancet Child & Adolescent Health*, *4*(8), 634–640. 10.1016/S2352-4642(20)30186-3 10.1016/S2352-4642(20)30186-3 PMC7292584 32540024

[CR30] O’Sullivan, K., Clark, S., McGrane, A., Rock, N., Burke, L., Boyle, N., Joksimovic, N., & Marshall, K. (2021). A qualitative study of child and adolescent mental health during the COVID-19 pandemic in Ireland. *International Journal of Environmental Research and Public Health*. 10.3390/ijerph18031062 33504101 10.3390/ijerph18031062 PMC7908364

[CR31] Panjwani, A. A., Bailey, R. L., & Kelleher, B. L. (2021). COVID-19 and behaviors in children with autism spectrum disorder: Disparities by income and food security status. *Research in Developmental Disabilities*. 10.1016/j.ridd.2021.104002 34147945 10.1016/j.ridd.2021.104002 PMC8276948

[CR32] Patel, J. A., Badiani, A. A., Nielsen, F. B. H., Assi, S., Unadkat, V., Patel, B., Courtney, C., & Hallas, L. (2020). COVID-19 and autism: Uncertainty, distress and feeling forgotten. *Public Health in Practice*. 10.1016/j.puhip.2020.100034 34173571 10.1016/j.puhip.2020.100034 PMC7392884

[CR33] Pellicano, E., Brett, S., den Houting, J., Heyworth, M., Magiati, I., Steward, R., Urbanowicz, A., & Stears, M. (2021). COVID-19, social isolation and the mental health of autistic people and their families: A qualitative study. *Autism,**26*(4), 914–927. 10.1177/13623613211035936 34362263 10.1177/13623613211035936

[CR34] Pereirinha, J. A. C., & Pereira, E. (2021). Social resilience and welfare systems under COVID-19: A European comparative perspective. *Global Social Policy,**21*(3), 569–594. 10.1177/14680181211012946

[CR35] Rothe, J., Buse, J., Uhlmann, A., Bluschke, A., & Roessner, V. (2021). Changes in emotions and worries during the Covid-19 pandemic: an online-survey with children and adults with and without mental health conditions. *Child and Adolescent Psychiatry and Mental Health*. 10.1186/S13034-021-00363-9 33612122 10.1186/s13034-021-00363-9 PMC7897360

[CR36] Scheeren, A. M., Howlin, P., Pellicano, L., Magiati, I., & Begeer, S. (2022). Continuity and change in loneliness and stress during the COVID-19 pandemic: A longitudinal study of autistic and non-autistic adults. *Autism Research,**15*(9), 1621–1635. 10.1002/aur.2787 35930166 10.1002/aur.2787 PMC9538450

[CR37] Seneta, E. (2013). A tricentenary history of the Law of Large Numbers. *Bernouilli,**19*(4), 1088–1121. 10.3150/12-bejsp12

[CR38] Shephard, E., Bedford, R., Milosavljevic, B., Gliga, T., Jones, E. J. H., Pickles, A., Johnson, M. H., Charman, T., BASIS Team. (2018). Early developmental pathways to childhood symptoms of attention-deficit hyperactivity disorder, anxiety and autism spectrum disorder. *Journal of Child Psychology and Psychiatry*. 10.1111/jcpp.12947 29963709 10.1111/jcpp.12947 PMC6694009

[CR39] Siracusano, M., Segatori, E., Riccioni, A., Gialloreti, L. E., Curatolo, P., & Mazzone, L. (2021). The impact of COVID-19 on the adaptive functioning, behavioral problems, and repetitive behaviors of Italian children with autism spectrum disorder: An observational study. *Children*. 10.3390/children8020096 33540683 10.3390/children8020096 PMC7913091

[CR40] Spinelli, M., Lionetti, F., Setti, A., & Fasolo, M. (2021). Parenting stress during the COVID-19 outbreak: Socioeconomic and environmental risk factors and implications for children emotion regulation. *Family Process,**60*(2), 639–653. 10.1111/famp.12601 32985703 10.1111/famp.12601

[CR41] Toseeb, U., & Asbury, K. (2023). A longitudinal study of the mental health of autistic children and adolescents and their parents during COVID-19: Part 1, quantitative findings. *Autism*. 10.1177/13623613221082715 35669991 10.1177/13623613221082715 PMC9805925

[CR42] van de Groep, S. I., Zanolie, K. I., Green, K. H., Sweijen, S. W., & Crone, E. A. (2020). A daily diary study on adolescents’ mood, empathy, and prosocial behavior during the COVID-19 pandemic. *PLOS One*. 10.1371/journal.pone.0240349 33027308 10.1371/journal.pone.0240349 PMC7540854

[CR43] van Steensel, F. J. A., Bögels, S. M., & Perrin, S. (2011). Anxiety Disorders in Children and Adolescents with Autistic Spectrum Disorders: A Meta-Analysis. *Clinical child and family psychology review*. 10.1007/s10567-011-0097-0 21735077 10.1007/s10567-011-0097-0 PMC3162631

[CR44] van Widenfelt, B. M., Goedhart, A. W., Treffers, P. D. A., & Goodman, R. (2003). Dutch version of the Strengths and Difficulties Questionnaire (SDQ). *European Child & Adolescent Psychiatry,**12*, 281–289. 10.1007/s00787-003-0341-3 14689260 10.1007/s00787-003-0341-3

[CR45] Vasa, R. A., Singh, V., Holingue, C., Kalb, L. G., Jang, Y., & Keefer, A. (2021). Psychiatric problems during the COVID-19 pandemic in children with autism spectrum disorder. *Autism Research: Official Journal of the International Society for Autism Research,**14*(10), 2113–2119. 10.1002/aur.2574 34231323 10.1002/aur.2574 PMC8420610

