# Supplementary material for: Psychosocial Outcomes in Autistic Children Before and During the COVID-19 Pandemic
Source: J Autism Dev Disord. 2023 Sep 10;54(10):3670–83. doi: 10.1007/s10803-023-06101-8 (PMC11461758; doi:10.1007/s10803-023-06101-8)
Supplement: Supplementary file 1 — Supplementary file1 (DOCX 24 kb) [file 10803_2023_6101_MOESM1_ESM.docx]

# Appendix A

# Full Results for Psychosocial Outcomes at Baseline

**Table A1**

*Differences for SDQ and AQ Scores at T0 (pre-COVID-19) Between Boys and Girls*

| **Domain** | **Boys** | | **Girls** | |  |
| --- | --- | --- | --- | --- | --- |
|  | ***M*** | ***SD*** | ***M*** | ***SD*** | ***p*** |
| SDQ Total | 17.46 | 5.92 | 19.15 | 6.33 | .221 |
| AQ Total | 81.68 | 11.02 | 81.54 | 10.24 | .930 |

**Table A2**

*Subdomain Correlations Within and Between SDQ and AQ at T0 (pre-COVID-19)*

|  |  | | AQ | | | | | | SDQ | | | | | |
| --- | --- | --- | --- | --- | --- | --- | --- | --- | --- | --- | --- | --- | --- | --- |
|  | Domain | | SOS | ROT | SWI | IMA | NMB | SOB | TOT | EMP | COP | HYP | PEP | PRO |
| AQ | | TOTa | .72 | .77 | .72 | .78 | .49 | .95 | .35 | .41 | .07 | 0 | .43 | -0.35 |
|  |  | SOS |  | .56 | .48 | .33 | .11 | .72 | .24 | .36 | .04 | -.17 | .38 | -.31 |
|  |  | ROT |  |  | .63 | .49 | .16 | .81 | .29 | .4 | .02 | 0 | .3 | -.13 |
|  |  | SWI |  |  |  | .53 | .09 | .78 | .47 | .47 | .14 | .16 | .41 | -.27 |
|  |  | IMA |  |  |  |  | .24 | .8 | .27 | .21 | .07 | .13 | .3 | -.25 |
|  |  | NMB |  |  |  |  |  | .23 | .05 | .08 | -.02 | -.08 | .15 | -.22 |
|  |  | SOB |  |  |  |  |  |  | .4 | .43 | .08 | .07 | .43 | -.31 |
| SDQ | | TOTs |  |  |  |  |  |  |  | .75 | .64 | .67 | .53 | -.44 |
|  |  | EMP |  |  |  |  |  |  |  |  | .32 | .28 | .22 | -.21 |
|  |  | COP |  |  |  |  |  |  |  |  |  | .31 | .14 | -.29 |
|  |  | HYP |  |  |  |  |  |  |  |  |  |  | .11 | -.24 |
|  |  | PEP |  |  |  |  |  |  |  |  |  |  |  | -.44 |

*Note*. TOTa: AQ Total Score, SOS: AQ Social Satisfaction, ROT: AQ Routine, SWI: AQ Switching, IMA: AQ Imagination, NMB: AQ Numbers and Patterns, SOB: AQ Social Behavior TOTs: SDQ Total Difficulties, EMP: SDQ Emotional Problems, COP: SDQ Conduct Problems, HYP: SDQ Hyperactivity.

**Table A3**

*Regression Coefficients of each SDQ Domain as Predicted by Parental Key Profession Status*

| SDQ domain | Predictor | B | SE | p | R^2^ |
| --- | --- | --- | --- | --- | --- |
| Total | Parent 1 | -2.51 | 1.37 | 0.0713 | 0.04 |
| Total | Parent 2 | 0.85 | 1.73 | 0.625 | 0.04 |
| Emotional Problems | Parent 1 | -1.34 | 0.63 | 0.0364 | 0.06 |
| Emotional Problems | Parent 2 | 1.17 | 0.85 | 0.175 | 0.03 |
| Hyperactivity | Parent 1 | -0.20 | 0.58 | 0.736 | 0.00 |
| Hyperactivity | Parent 2 | -0.32 | 0.72 | 0.663 | 0.00 |
| Peer Problems | Parent 1 | -0.49 | 0.51 | 0.339 | 0.01 |
| Peer Problems | Parent 2 | 0.06 | 0.64 | 0.932 | 0.00 |
| Prosocial Behavior | Parent 1 | -0.73 | 0.64 | 0.259 | 0.02 |
| Prosocial Behavior | Parent 2 | -0.17 | 0.78 | 0.825 | 0.00 |
| Conduct Problems | Parent 1 | -0.49 | 0.46 | 0.2893 | 0.02 |
| Conduct Problems | Parent 2 | -0.05 | 0.59 | 0.9298 | 0.00 |

# Appendix B

# Selected Model 5 Results for Separate SDQ Subdomains

**Table B1**

*Fixed Effects of Selected Final Multilevel Model; Outcome: SDQ Difficulty Score per Subdomain*

| **SDQ Domain** | **Effect** | ***FE Estimate*** | ***SE*** | ***p*** |
| --- | --- | --- | --- | --- |
| Total Score | Time  Type of Education  Gender  Age | -1.23  1.33  1.64  -0.32 | 0.20  0.53  0.70  0.08 | < .001  .013  .020  < .001 |
| Emotional Problems | Time  Type of Education  Gender  Age | 0.02  -0.02  1.64  -0.02 | 0.09  0.26  0.35  0.04 | .83  .94  < .001  .59 |
| Conduct Problems | Time  Type of Education  Gender  Age | -0.21  0.02  0.37  -0.10 | 0.07  0.17  0.22  0.02 | .001  .91  .10  < .001 |
| Hyperactivity | Time  Type of Education  Gender  Age | -0.89  0.72  -0.27  -0.15 | 0.09  0.22  0.28  0.03 | < .001  .001  .33  < .001 |
| Peer Problems | Time  Type of Education  Gender  Age | -0.15  0.66  -0.11  -0.04 | 0.08  0.20  0.26  0.03 | .045  < .001  .68  .16 |
| Prosocial Behavior | Time  Type of Education  Gender  Age | 0.21  -0.26  0.15  0.10 | 0.09  0.23  0.32  0.03 | .014  .28  .63  .004 |

# Appendix C

# AQ and SDQ Reliabilities for Total and Subdomain Scores

**Table C1**

*Reliabilities for AQ (Cronbach’s α and McDonald’s ω)*

Autism-Spectrum Quotient

|  | α | ω |
| --- | --- | --- |
| AQ Total score | .85 | .85 |
| AQ Social | .74 | .75 |
| AQ Routine | .67 | .67 |
| AQ Switching | .58 | .60 |
| AQ Imagination | .70 | .70 |
| AQ Numbers and Patterns | .71 | .74 |

**Table C2**

*Reliabilities for SDQ (Cronbach’s α and McDonald’s ω)*

Strengths and Difficulties Questionnaire

| SDQ domain | **T0** | | | | **T1** | | | | **T2** | | | |  |
| --- | --- | --- | --- | --- | --- | --- | --- | --- | --- | --- | --- | --- | --- |
|  | | α | | ω | | α | | ω | | α | | ω | |
| SDQ Total score | .80 | | .80 | | .73 | | .74 | | .78 | | .78 | |  |
| SDQ Emotional Problems | .79 | | .80 | | .78 | | .78 | | .76 | | .77 | |  |
| SDQ Conduct Problems | .62 | | .63 | | .60 | | .64 | | .62 | | .63 | |  |
| SDQ HyperActivity | .73 | | .73 | | .59 | | .62 | | .63 | | .67 | |  |
| SDQ Peer Problems | .52 | | .54 | | .55 | | .55 | | .57 | | .57 | |  |
| SDQ Prosocial Behavior | .79 | | .80 | | .76 | | .76 | | .77 | | .78 | |  |
